# Editorial: Innovative diagnostic and therapeutic strategies for neuroendocrine tumors: a multidisciplinary approach

**DOI:** 10.3389/fonc.2026.1939733

**Published:** 2026-07-24

**Authors:** Patricia Borges de Souza, Matteo Zurlo, Sara Bravaccini, Martina Rosticci, Massimiliano Mazza, Maria Chiara Zatelli

**Affiliations:** 1 IRCCS Istituto Romagnolo per lo Studio dei Tumori (IRST) "Dino Amadori", Meldola, Italy; 2 Department of Medicine and Surgery, University of Enna “Kore”, Enna, Italy; 3 Department of Medical Sciences, Section of Endocrinology, Geriatrics and Internal Medicine, University of Ferrara, Ferrara, Italy

**Keywords:** neuroendocrine neoplasia, precision medicine, radiomics, SHAP (SHapley Additive exPlanations) analysis, SSTR, somatostatin receptor

Neuroendocrine neoplasms (NENs) comprise a heterogeneous family of tumors arising from neuroendocrine cells throughout the body. Although historically considered rare, their incidence and prevalence have steadily increased over recent decades owing to improvements in diagnostic imaging, pathology, and clinical awareness (1, 2). This growing recognition has been accompanied by major advances in molecular imaging, surgery, systemic therapies, and peptide receptor radionuclide therapy, all of which have significantly improved patient management (3, 4). Nevertheless, NENs still present substantial clinical challenges. Their remarkable biological heterogeneity translates into highly variable clinical behavior, prognosis, and treatment response, making personalized management an important yet still incompletely achieved objective (5).

In this context, this Research Topic was conceived to highlight innovative diagnostic and therapeutic strategies to advance precision medicine in NENs. Although the contributions address diverse clinical and translational questions, they converge on a common theme: improving patient stratification through a better understanding of tumor biology and by exploiting emerging technologies for individualized clinical decision-making. Collectively, the accepted articles illustrate how advances in imaging, computational analysis, molecular pharmacology, and multidisciplinary clinical management are progressively reshaping the field of neuroendocrine oncology.

A prominent theme emerging from this Research Topic is the evolving role of imaging. Traditionally regarded as a tool for tumor detection and staging, imaging is increasingly becoming a quantitative biomarker capable of predicting tumor behavior and therapeutic response. This evolution reflects the broader transition toward precision oncology, in which imaging contributes not only anatomical information but also functional and biological insights (6, 7).

Two studies in this Research Topic demonstrate the growing contribution of radiomics and artificial intelligence (AI) to NENs management. Kong et al. developed an interpretable machine-learning model capable of accurately distinguishing aggressive from non-aggressive pancreatic NENs using routinely acquired contrast-enhanced CT features. Importantly, the use of SHapley Additive exPlanations (SHAP) provided transparent interpretation of model predictions, addressing one of the major barriers to AI clinical adoption. Rather than functioning as a “black box,” the model allows clinicians to identify peculiar imaging characteristics contributing to each prediction, thereby increasing confidence in AI-assisted decision making.

Complementing this work, Feng et al. explored the use of radiomics to predict therapeutic response. By integrating quantitative CT-derived features with clinical parameters, including Ki-67 index and metastatic burden, the Authors developed a nomogram capable of identifying patients more likely to benefit from surufatinib therapy. Together, these studies exemplify the growing role of radiomics as a non-invasive biomarker that captures tumor heterogeneity and supports treatment personalization. Importantly, both investigations emphasize that computational approaches achieve their greatest value when integrated with conventional clinical and biological information rather than replacing them.

Functional imaging likewise continues to provide novel biological insights beyond lesion localization. Kunte et al. demonstrated that physiological pancreatic uptake of Somatostatin receptor (SSTR)-targeting radiotracers is significantly influenced by glucose metabolism and antidiabetic therapy. These findings suggest that metabolic status should be considered when interpreting pancreatic SSTR PET/CT examinations, since altered glucose homeostasis may affect background pancreatic uptake and consequently influence lesion detectability. Beyond its practical implications for image interpretation, this study illustrates the intimate relationship between endocrine physiology, metabolism, and molecular imaging, emphasizing that imaging biomarkers cannot be interpreted independently of the biological environment in which they are generated.

While advances in imaging improve patient stratification, the development of more effective therapies remains equally critical. SSTR ligands (SRLs) remain the cornerstone of systemic therapy for well-differentiated NENs because of their well-established antisecretory and antiproliferative effects. However, differences in SSTR subtype expression, particularly the variable SSTR2 and SSTR5 co-expression, together with emerging resistance mechanisms, prompted the development of next-generation receptor-targeted compounds. Recent reviews highlight how advances in receptor pharmacology and structural biology are driving the design of novel SRLs with improved receptor selectivity and therapeutic potential (8). In this regard, Fedeli et al. provide compelling preclinical evidence supporting the development of dual SSTR2/SSTR5 agonists as next-generation therapeutic agents. Using both human and murine NEN models, the authors demonstrated enhanced antisecretory and antiproliferative activity compared with currently available first-generation somatostatin analogues. These findings reinforce the concept that a more comprehensive understanding of receptor biology can drive the rational design of new therapies capable of overcoming some of the limitations of existing treatments.

Precision medicine, however, extends beyond technological innovation and targeted therapies. It also requires improving the diagnosis and management of rare clinical entities for which evidence remains scarce. Puliani et al. address this need by presenting a case series together with a comprehensive review of middle ear NENs, one of the rarest manifestations of neuroendocrine disease. Their work highlights the diagnostic complexity of these lesions and underscores the importance of integrating pathology, imaging, surgery, nuclear medicine, and systemic treatment within a multidisciplinary framework. Such contributions are particularly valuable in rare diseases, where prospective clinical trials are difficult to conduct and carefully documented clinical experience remains an essential source of evidence.

Taken together, the studies included in this Research Topic highlight several important directions for future research. First, imaging is evolving into a multidimensional biomarker that combines anatomical, functional, and computational information to improve diagnosis, prognosis, and treatment selection. Second, advances in tumor biology are enabling the development of increasingly selective therapeutic strategies targeting specific molecular pathways and receptor profiles. Finally, the successful implementation of precision medicine will require integrating imaging, molecular biomarkers, AI, and clinical data into comprehensive decision-support systems capable of reflecting NEN biological complexity. Achieving this objective will depend on multidisciplinary collaboration among clinicians, radiologists, nuclear medicine physicians, pathologists, molecular biologists, and computational scientists, as well as on prospective multicenter validation of emerging biomarkers and predictive models.

In conclusion, the contributions collected in this Research Topic demonstrate the rapid evolution of neuroendocrine oncology toward a more personalized and biologically informed approach to patient care. By advancing quantitative imaging, computational modeling, receptor-directed therapeutics, and multidisciplinary management of rare disease entities, these studies contribute important pieces to the evolving landscape of precision medicine in NENs. We hope that this Research Topic will stimulate further collaborative research and accelerate the translation of these innovations into improved outcomes for patients with NENs.
